# Persistent dyslipidemia in treatment of lysosomal acid lipase deficiency

**DOI:** 10.1186/s13023-020-1328-6

**Published:** 2020-02-24

**Authors:** Amanda Barone Pritchard, Alanna Strong, Can Ficicioglu

**Affiliations:** 1 Division of Human Genetics and Metabolism, Children’s Hospital of Philadelphia, 3401 Civic Center Blvd, Philadelphia, PA 19104 USA; 2 Present address: C.S. Mott Children’s Hospital, Michigan Medicine, 1500 E Medical Center Dr, Ann Arbor, MI 48109 USA

**Keywords:** Lysosomal acid lipase deficiency, Sebelipase alfa, Hypercholesterolemia, Enzyme replacement therapy

## Abstract

**Background:**

Lysosomal acid lipase deficiency (LALD) is an autosomal recessive inborn error of lipid metabolism characterized by impaired lysosomal hydrolysis and consequent accumulation of cholesteryl esters and triglycerides. The phenotypic spectrum is diverse, ranging from severe, neonatal onset failure to thrive, hepatomegaly, hepatic fibrosis, malabsorption and adrenal insufficiency to childhood-onset hyperlipidemia, hepatomegaly, and hepatic fibrosis. Sebelipase alfa enzyme replacement has been approved by the Food and Drug Administration for use in LALD after demonstrating dramatic improvement in transaminitis and dyslipidemia with initiation of enzyme replacement therapy.

**Methods:**

A chart review was performed on 2 patients with childhood-onset, symptomatic LALD with persistent dyslipidemia despite appropriate enzyme replacement therapy to identify biological pathways and risk factors for incomplete response to therapy.

**Results:**

Two patients with attenuated, symptomatic LALD had resolution of transaminitis on enzyme replacement therapy without concomitant effect on dyslipidemia despite dose escalation and no evidence of antibody response to enzyme.

**Conclusion:**

Enzyme replacement therapy does not universally resolve all complications of LALD. Persistent dyslipidemia remains a clinically significant issue, likely related to the complex metabolic pathways implicated in LALD pathogenesis. We discuss the possible mechanistic basis for this unexpected finding and the implications for curative LALD therapy.

## Background

Lysosomal acid lipase deficiency (LALD, Online Mendelian Inheritance in Man number 278000) is a rare, autosomal recessive inborn error of lipid metabolism caused by biallelic pathogenic variants in the *LIPA* gene, which encodes the enzyme lysosomal acid lipase (LAL). The disease spectrum is diverse, ranging from the historically described infantile Wolman Disease to the later-onset cholesteryl ester storage disease (CESD). Wolman Disease is characterized by hepatosplenomegaly, cholestasis, hepatic fibrosis, failure to thrive, malabsorption, and adrenal insufficiency, and was universally fatal without bone marrow transplantation prior to the arrival of enzyme replacement therapy [1]. CESD typically presents during mid-childhood to late adulthood with hepatomegaly with or without splenomegaly, transaminitis, cirrhosis, hyperlipidemia and atherosclerotic cardiovascular disease [2]. Liver pathology in LALD is caused by the lysosomal accumulation of cholesteryl esters and triglycerides due to their impaired hydrolysis from decreased or absent LAL activity. The consequent deficiency in cellular free fatty acids and free cholesterol from impaired hydrolysis leads to activation of the sterol response element-binding protein (SREBP) pathway, which increases de novo cholesterol biosynthesis [3]. Concomitant inhibition of the liver X receptor (LXR) pathway decreases cholesterol efflux and high-density lipoprotein (HDL) generation, leading to the dyslipidemia characteristic of this disease - specifically, elevated low-density lipoprotein cholesterol, hypertriglyceridemia, and decreased high-density lipoprotein cholesterol [4, 5].

Treatment for LALD was historically supportive until 2015 with Food and Drug Administration (FDA) approval of enzyme replacement therapy (ERT). The recombinant enzyme, sebelipase alfa, is given once every 2 weeks as an intravenous infusion and is taken up by target tissues, predominantly the liver, via the mannose-6-phosphate receptor [6]. A Phase III trial of biweekly enzyme replacement for 52 weeks demonstrated dramatic clinical and biochemical improvement, including normalization or near-normalization of alanine aminotransferase (ALT) levels, improved lipid parameters including reduction in low-density lipoprotein cholesterol (LDL-C) and triglycerides and increased high-density lipoprotein cholesterol (HDL-C) levels, and decreased hepatic fat content [7].

Given the pathophysiology of disease, lifelong ERT should lead to sustained improvement in liver pathology and lipoprotein profile. We report two patients with LALD previously reported as part of the initial ERT clinical trial [6] who had stable improvement in ALT and AST without improvement in dyslipidemia while receiving enzyme replacement, and discuss potential mechanistic explanations for this unpredicted finding.

## Results

### Clinical presentation and laboratory data patient A

**Fig. 1:**
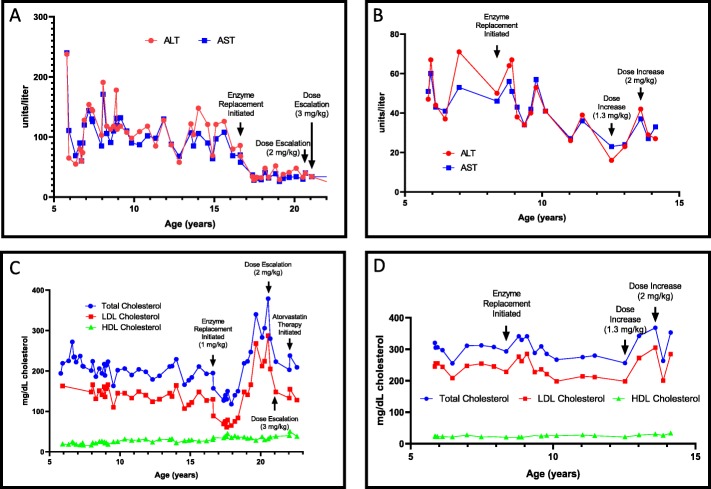
Patient clinical data **a**) Graph of transaminase levels for Patient A prior to and with initiation and escalation of enzyme replacement therapy **b**) Graph of transaminase levels for Patient B prior to and with initiation and escalation of enzyme replacement therapy **c**) Graph depicting total cholesterol, LDL cholesterol and HDL cholesterol levels for Patient A prior to and with initiation and escalation of enzyme replacement therapy **d**) Graph depicting total cholesterol, LDL cholesterol and HDL cholesterol levels for Patient B prior to and with initiation and escalation of enzyme replacement therapy Abbreviations: ALT: alanine aminotransferase; AST: aspartate transaminase; HDL: high-density lipoprotein; LDL: low-density lipoprotein

Patient A presented at 5 years of age with significant hepatosplenomegaly. Laboratories at that time were notable for elevated total cholesterol (219 mg/dL, normal < 182 mg/dL), elevated LDL-C (163 mg/dL, normal < 140 mg/dL), decreased HDL cholesterol (19 mg/dL, normal > 35 mg/dL), hypertriglyceridemia (183 mg/dL, normal < 125 mg/dL), and transaminitis (ALT 65 and AST 111 U/L, normal 50 U/L). Liver biopsy was notable for hepatocytes with triglyceride droplets and macrophages containing cholesterol crystals, concerning for cholesteryl ester storage disease. Confirmatory enzyme testing was diagnostic (blood enzyme activity 0.007 nmol/punch/gram; affected < 0.009), and genetic testing was notable for one copy of the classically described exon-skipping mutation c.894 G > A (p.Q298=) and an exon 4 deletion resulting in a frameshift. She was subsequently managed with a low-fat (less than 50 g of fat daily, 25% of total calories) and low-cholesterol diet (less than 130 mg daily), simvastatin (80 mg daily), and vitamin K. She continued to develop normally and grow normally, with her height tracking in the 25th% and her weight tracking in the 50th – 75th%. She had stellar academic performance. LDL cholesterol and triglyceride levels remained elevated, HDL remained low, and transaminases remained elevated (Fig. 1a, c).

At 16 years and 7 months of age sebelipase alfa (1 mg/kg biweekly) therapy was developed and was added to her treatment regimen, initially as research as part of the clinical trial, and subsequently on a clinical basis when the FDA approved sebelipase alfa. Simvastatin therapy was continued throughout her time in the clinical trial. Though there was initial improvement in her dyslipidemia on enzyme replacement therapy, her lipid levels soon rebounded. Her dyslipidemia worsened when statin therapy was discontinued at 19 years of age. Dose escalation to 2 mg/kg biweekly was attempted starting at 20 years and 8 months of life to improve her lipid parameters with no effect. Dose escalation to 3 mg/kg was also attempted at 21 years and 1 month of life, also without success (Fig. 1c). Kidney and thyroid function were checked and were normal as was a pancreatic ultrasound. Genetic testing for familial hypercholesterolemia (Gene Dx sequencing panel including the *APOB, LDLR, LDLRAP1,* and *PCSK9* genes) did not reveal any suggestive variants to explain her persistent dyslipidemia. Antibody studies were sent and were negative. Of note, despite adherence to her low-fat, low-cholesterol diet, patient experienced significant weight gain between the ages of 18 and 20 years, with a body mass index in the ninety-second percentile. Low dose statin therapy (atorvastatin 10 mg daily) was re-initiated at 22 years of age; with improvement and normalization of her lipid profile (Fig. 1c). She continues to have elevated oxysterols, moderate to severe hepatosplenomegaly (current liver volume 2648 mL) with MRI evidence of steatosis (9% fat content) and increased liver and spleen stiffness (3.4 kPa and 6.9 kPa, respectively, normal < 2.9 kPa). Her hepatosplenomegaly has continued to worsen on enzyme replacement therapy.

### Clinical presentation and laboratory data patient B

Patient B came to medical attention at 5 years of age due to hepatomegaly and persistent low-grade fevers. She underwent a liver biopsy and pathology was consistent with cholesteryl ester storage disease. Subsequently obtained laboratories were notable for elevated total cholesterol (320 mg/dL, normal < 170 mg/dL), elevated LDL-C (245 mg/dL, normal < 110 mg/dL), decreased HDL cholesterol (23 mg/dL, normal > 37 mg/dL), hypertriglyceridemia (260 mg/dL, normal < 115 mg/dL), and transaminitis (ALT 47 and AST 51 U/L, normal 24 and 39 U/L, respectively). *LIPA* gene sequencing revealed compound heterozygous pathogenic variants, a novel c.57_60delTGAG (p.E20fsX6) mutation and the classically described exon-skipping mutation c.894G > A (p.Q298=).

She adhered to a low-fat diet (40 g fat daily, 20% of total calories) and low-cholesterol diet (less than 160 mg daily), and at 8 years of age was started on sebelipase alfa therapy as part of the clinical trial. She continued on ERT through the open-label trial and was transitioned to commercial enzyme therapy at 1 mg/kg biweekly once ERT was FDA approved. Her hepatomegaly and transaminitis resolved (Fig. 1b); however, due to her persistent dyslipidemia, her ERT dose was increased to 2 mg/kg biweekly. This did not improve her lipid parameters (Fig. 1d). Thyroid testing, pancreatic ultrasound and renal function were normal, and she reported good adherence to her low-fat, low-cholesterol diet. A familial dyslipidemia panel was notable only for her known bilallelic *LIPA* pathogenic variants (panel genes: *ABCA1, ABCG5, ABCG8, ANGPTL3, APOA1, APOA5, APOB, APOC2, APOC3, APOE, CETP, CYP27A1, CYP7A1, GCKR, GPD1, GPIHBP1, LCAT, LDLR, LDLRAP1, LIPA, LIPC, LMF1, LPL, MTTP, PCSK9, SAR1B, SCARB1, and STAP1)*. Of note, menarche was at 12.5 years of age, nearly concurrent with the acute worsening of her dyslipidemia. She is otherwise growing and developing well, with her height tracking consistently in the 20th – 30th% and her weight consistently in the 50th%. Her hepatosplenomegaly had resolved with enzyme replacement therapy and she never had evidence of increased liver stiffness (current 2.1 kPa, normal < 2.9 kPa). She continues to have elevated oxysterols and dyslipidemia, but her hepatic fat content has remained stable at 9%. Statin therapy was not initiated due to parental preference.

## Discussion

LALD is an extremely rare lysosomal storage disorder characterized by hepatosplenomegaly, dyslipidemia, hepatic dysfunction with progression to fibrosis and cirrhosis, and predisposition to atherosclerotic cardiovascular disease. Disease severity largely reflects residual enzyme activity, with complete absence of lysosomal acid lipase activity causing neonatal onset disease, and partial enzyme deficiency associated with later-onset symptomatology and a more chronic course [8]. The development and approval of ERT, sebelipase alfa, has dramatically modified the natural history of this uncommon diagnosis, prolonging the lives of children with neonatal onset disease and improving outcomes and quality of life for individuals with later-onset disease [6, 7, 9, 10].

Interestingly, response to enzyme replacement is neither universal nor uniform with regard both to liver metrics and dyslipidemia [7]. Most individuals receiving ERT experience dramatic improvements in lipid profile; however, there have been several cases of incomplete response and even worsening dyslipidemia on treatment [5, 10–12]. The etiology of this variability is unknown. Here we describe in detail 2 patients with childhood-onset LALD who had little to no sustained improvement in lipid parameters on appropriate therapy. We highlight these cases as a mechanism to better understand the pathophysiology of LALD and inform an effective treatment for residual disease in therapy non-responders.

The simplest explanation for lack of response to ERT is emergence of an enzyme-specific neutralizing antibody, which is a common phenomenon in patients treated with enzyme replacement [13, 14]. Though anti-drug antibodies are more commonly reported in neonatal-onset disease associated with complete enzyme deficiency, as the ERT is then a foreign enzyme to the body, antibody production has been reported in individuals with residual enzyme activity. Experiences with antibodies reported to date in LALD patients have been relatively innocuous, suggesting that these antibodies are often transient and do not interfere with the safety or efficacy of the enzyme replacement [6, 7, 10, 15]. There is, however, a single report of a child who experienced significant blunting of response to therapy with emergence of a high antibody titer [10]. Importantly, patients with anti-drug antibodies usually have initial response to therapy followed by reversion to pre-treatment pathology, in contrast to our patients, whose lipid profiles consistently showed minimal response to therapy. Additionally, emergence of an antibody should interfere with all facets of the therapeutic benefit of ERT, both liver pathology and dyslipidemia. It is difficult to envision a mechanism by which an antibody would interfere with correction of the dyslipidemia alone, as is seen in our patients. Additionally, both of our patients had negative antibody testing.

Another possible explanation relates to a global shortcoming of enzyme replacement therapy: enzyme is taken up by tissues with the greatest access and highest affinity receptors, not necessarily the tissue with the greatest physiological need. This phenomenon is exemplified by the experience with ERT for Fabry Disease, which therapeutically requires enzyme delivery to the endothelium, heart and kidney, but instead has preferential hepatic uptake, limiting its delivery to these crucial tissues [16, 17]. Similarly, sebelipase alfa is taken up predominantly by liver, though other tissues require enzyme, and these tissues may continue to upregulate de novo cholesterol biosynthesis and contribute to dyslipidemia. In conflict with this potential paradigm, animal studies suggest that the liver is the most physiologically important contributor to the increased de novo cholesterol biosynthesis in LALD [18]. Indeed, liver-specific *LIPA* deficiency is associated with dyslipidemia and increased cholesteryl ester storage [19]. Restoration of LAL activity through virus-mediated transduction or transgenics in mouse models of LALD and liver-transplantation in LALD patients has proven beneficial [11, 20–24]. Of course, both gene replacement and liver transplantation create a sustained reservoir of LAL-producing cells, capable of widely distributing enzyme for uptake into all tissues via the mannose-6-phosphate receptor. ERT, however, provides intermittent availability of enzyme with limited cell distribution.

**Fig. 2:**
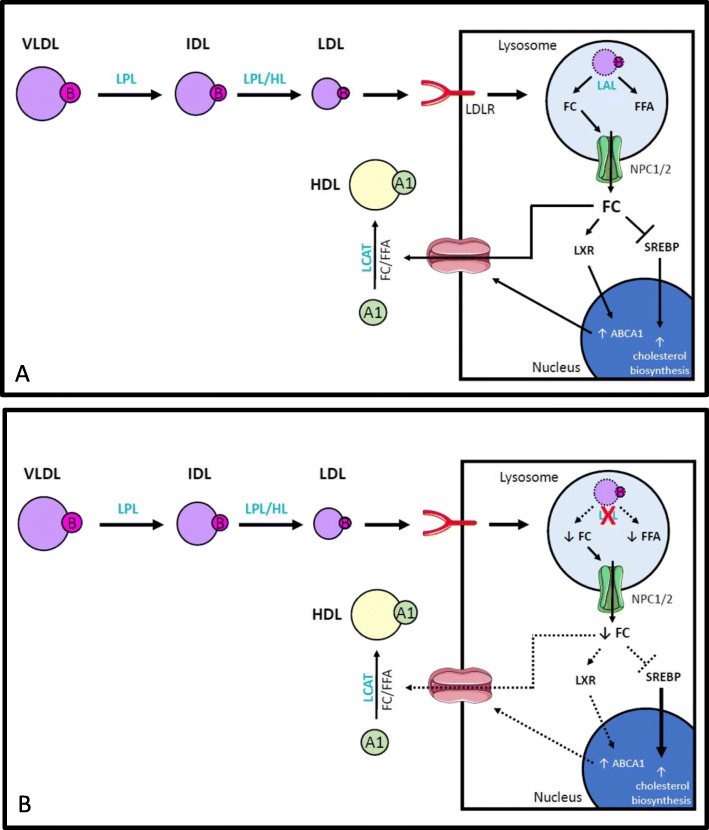
Lipoprotein metabolism dysregulation in lysosomal acid lipase deficiency **a**) The liver secretes VLDL, which is lipolyzed in the periphery by LPL and HL to generate LDL. LDL is taken up via the LDLR and is trafficked to the lysosome for degradation. LAL hydrolyzes the cholesteryl esters and triglycerides in LDL to FFA and FC. FC prevents SREBP pathway activation, thereby decreasing de novo cholesterol biosynthesis. FC also activates the LXR pathway to increase ABCA1 expression. ABCA1 effluxes FC to an APOA1 acceptor. The FC is esterified by LCAT to form the cholesteryl ester in HDL **b**) In LALD, inability to hydrolyze LDL cholesteryl ester and triglyceride impairs FFA and FC generation. Decreased FC generation results in increased SREBP pathway activation, which increases de novo cholesterol biosynthesis. Decreased FC also decreases LXR pathway activation, which decreases ABCA1 expression and impairs HDL formation. Accumulation of the FC and FFAs in the lysosome causes hepatic, adrenal, and intestinal toxicity. Abbreviations: APOA1: Apolipoprotein A1; ABCA1: ATP-binding cassette transporter-1; FC: Free cholesterol; FFA: Free fatty acid; HL: Hepatic lipase; HDL-C: High-density lipoprotein cholesterol; LCAT: Lecithin-cholesterol acyltransferase; LPL: Lipoprotein lipase; LXR: Liver X receptor; LDL: Low-density lipoprotein cholesterol; LDLR: Low-density lipoprotein receptor; LAL: Lysosomal acid lipase; LALD: Lysosomal acid lipase deficiency; SREBP: Sterol regulatory element-binding protein; VLDL-C: Very-low density lipoprotein cholesterol

Lipoprotein metabolism is inherently complex and subject to multiple levels of regulation, and subtle genetically determined differences in lipid handling may explain the persistent dyslipidemia in a subset of LALD patients (Fig. 2). Dietary cholesterol absorption is accomplished through the intestinal transporter Niemann-Pick C1-Like 1 (NPC1L1). Subsequently, hepatic secretion of the LDL-precursor very low-density lipoprotein cholesterol (VLDL-C) is influenced by lipid availability and the efficiency of particle lipidation. The rate of LDL removal from circulation is then influenced by the rate of peripheral lipolysis by endothelial lipases and by cellular uptake via the LDL receptor and other endocytic receptors. The lysosomal efflux of hydrolyzed cholesterol is dependent on the Niemann-Pick C1/2 transport system. Meanwhile, the generation of high-density lipoprotein cholesterol is determined by the rate of ATP-binding cassette transporter-1 (ABCA1)-mediated cholesterol efflux, and the rate of HDL turnover and clearance is dictated by cholesteryl ester transfer protein activity and scavenger receptor type B class 1 kinetics [5]. This system remains in a tenuous balance. Though our patients had negative gene panel testing for many of the genes known to be associated with Mendelian hypercholesterolemia syndromes, these panels do not account for all modifier genes within this pathway nor for noncoding variants, alleles of small effect size, or polymorphisms in known genes [5, 25]. Importantly, common polymorphisms in all of these cholesterol metabolism genes are associated with population-based variation in lipoprotein profile and atherosclerotic cardiovascular disease risk, and individuals with LALD are not exempt from the effects of these variants [26–28]. Interestingly, ezetimibe, a pharmacological inhibitor of the NPC1L1 cholesterol transporter, has shown significant benefit in both animal models and patients with LALD, consistent with noncanonical LDL pathways modifying the LALD dyslipidemia phenotype [11, 29, 30].

There additionally may be baseline lipoprotein pathway dysregulation in LALD from a “metabolic imprint” due to developmental exposure to perceived cholesterol paucity. LALD patients have chronic SREBP pathway upregulation, which would be predicted to cause increased de novo cholesterol biosynthesis as well as increased LDL receptor-mediated LDL clearance. However, kinetic studies and protein analyses in humans suggest that LDLR activity is not universally increased in LALD, even with initiation of statin therapy, suggesting that individuals with LALD may have a disconnect between SREBP-mediated activation of de novo cholesterol biosynthesis and enhanced LDL uptake [5, 31–33]. Perhaps individuals with persistent dyslipidemia have restoration of SREBP-mediated downregulation of LDLR with ERT without the expected concomitant reduction in de novo cholesterol biosynthesis, a dangerous point of inflection on a dyslipidemia curve. These hypotheses are all theoretical at this time and will require additional work to clarify.

Beyond development, the artificial nature of ERT could adversely affect lipoprotein metabolism. LALD patients on ERT constantly alternate between a hydrolysis phase when enzyme is first supplied and a storage phase when enzyme is no longer available. This “feast to famine” phenomenon was demonstrated in the Phase III clinical trials for sebelipase alfa. Baseline dyslipidemia worsened with initiation of ERT, suspected to be secondary to increased substrate for VLDL secretion and exacerbation of the cholesterol efflux deficit due to sudden, overwhelming lysosomal triglyceride and cholesteryl ester hydrolysis. Perhaps individuals with persistent dyslipidemia are more sensitive to these fluctuations [7].

Specific to our patients, Patient A experienced rapid weight gain starting at 18 years of age, and obesity is a known risk factor for development of dyslipidemia. Patient B experienced menarche at 12.5 years of age, which closely parallels the time at which her dyslipidemia worsened. Menarche and puberty are known to affect lipid levels. These confounders considered, a fundamental lesson learned from these patients is that the threshold required to correct the dyslipidemia of LALD seems higher than that needed to protect hepatic integrity and correct the malabsorption phenotype. This would argue that lipid-lowering medications should have a central role in the treatment of LALD despite the advent of enzyme replacement therapy. The underlying biology of lipid metabolism is complex, and this is compounded in the context of LALD. Truly understanding the etiology of suboptimal response to ERT will require in vivo kinetic studies to assess LDL turnover, de novo cholesterol biosynthesis, hepatic lipoprotein secretion and cholesterol efflux. More thorough genetic profiling could be done to investigate variants in lipid genes that may be additionally modifying the lipid phenotype in LALD. The benefit of ezetimibe and statin therapy in improving the dyslipidemia in patients receiving and not receiving ERT adds credence to the importance of contributing pathways beyond lysosomal acid lipase to overall disease course. Further studies will be needed to clarify the exact nature of these influencing pathways. While these studies are in progress, historical experience suggests that anti-hyperlipidemia medications remain a powerful tool to improve plasma lipids in LALD patients who achieve insufficient benefit from ERT.

## Conclusion

The development of ERT for LALD has dramatically altered the natural history of this diagnosis; however, there remain incomplete responders with persistent dyslipidemia despite maximized ERT. Understanding the biology of lack of response to ERT will enable creation of better therapeutic options for this population and will also increase our understanding of the pathophysiology of this rare disease.

## Methods

Chart review was conducted on 2 patients with LALD with attention to dyslipidemia and response to enzyme replacement therapy. Both patients were previously reported as part of the initial ERT clinical trial [6]. Informed consent to use and publish this medical information was obtained from both patients and their parents. Both patients were medically managed prior to development of ERT. Both patients are followed by geneticists as well as metabolic dieticians. Enzyme testing and genetic testing was performed by Alexion as part the clinical trial; all other laboratories were done at The Children’s Hospital of Philadelphia.

## Data Availability

Not applicable.

## Abbreviations

ABCA1: ATP-binding cassette transporter-1
APOA1: Apolipoprotein A1
CESD: Cholesteryl ester storage disease
ERT: Enzyme replacement therapy
FC: Free cholesterol
FDA: Food and drug administration
FFA: Free fatty acid
HDL-C: High-density lipoprotein cholesterol
HL: Hepatic lipase
LAL: Lysosomal acid lipase
LALD: Lysosomal acid lipase deficiency
LCAT: Lecithin-cholesterol acyltransferase
LDL-C: Low-density lipoprotein cholesterol
LDLR: Low-density lipoprotein receptor
LPL: Lipoprotein lipase
LXR: Liver X receptor
NPC1L1: Niemann-Pick C1-Like 1
SREBP: Sterol regulatory element-binding protein
VLDL-C: Very-low density lipoprotein cholesterol
